# AAV9‐mediated AIRE gene delivery clears circulating antibodies and tissue T‐cell infiltration in a mouse model of autoimmune polyglandular syndrome type‐1

**DOI:** 10.1002/cti2.1166

**Published:** 2020-09-03

**Authors:** Sarah Almaghrabi, Mimoun Azzouz, Rachid Tazi Ahnini

**Affiliations:** ^1^ Department of Infection, Immunity and Cardiovascular Disease University of Sheffield Sheffield UK; ^2^ Faculty of Applied Medical Sciences King Abdulaziz University Jeddah Saudi Arabia; ^3^ Sheffield Institute for Translational Neuroscience (SITRaN) Department of Neuroscience The Medical School University of Sheffield Sheffield UK

**Keywords:** autoimmune polyglandular syndrome type‐1, adeno‐associated virus serotype 9, autoimmune regulator gene, intrathymic injection, gene therapy

## Abstract

**Objectives:**

Autoimmune polyglandular syndrome type‐1 (APS‐1) is a monogenic recessive disorder characterised by multiple endocrine abnormalities, chronic mucocutaneous candidiasis and high titres of serum autoantibodies. To date, no curative treatment is available; current therapies manage the symptoms rather than treating the cause and have major side effects. APS‐1 is caused by mutations in the autoimmune regulator (*AIRE*) gene. AIRE mediates central tolerance by directing the ectopic expression of tissue‐specific antigens (TSAs) in medullary thymic epithelial cells, causing the deletion of self‐reactive thymocytes. Therefore, loss‐of‐function mutations in *AIRE* result in a multisystem autoimmune disease. Because of the monogenic aetiology of APS‐1 and availability of an APS‐1 mouse model, we have explored the option of restoring functional AIRE using adeno‐associated virus serotype 9 (AAV9).

**Methods:**

The efficacy of AAV9‐AIRE (AAV9 carrying AIRE cDNA) gene therapy was assessed in an APS‐1 mouse model. We performed intrathymic injection of AAV9‐AIRE into APS‐1 mouse model using ultrasound imaging technique to accurately locating the thymus. We evaluated the efficiency of this approach alongside measures of autoimmunity and histology of target tissues.

**Results:**

Intrathymic injection of AAV9‐AIRE demonstrated high transduction efficiency and restored AIRE expression in the thymus. AIRE gene delivery led to a significant increase in TSA expression, and importantly a significant reduction of serum autoantibodies in treated versus control mice, which fell to near‐undetectable levels by 4 weeks post‐treatment. Furthermore, histological analysis of treated animals showed near‐normal tissue morphology with no lymphocytic infiltrations, a hallmark of untreated *Aire‐*deficient mice.

**Conclusion:**

This study has demonstrated the feasibility of AAV9‐AIRE as a vehicle for gene therapy for APS‐1.

## Graphical abstract

In a mouse model of APS‐1, direct AAV9‐AIRE injection into the thymus in pre‐symptomatic mice led to clearance and protection of all tissues targeted by the immune attack.

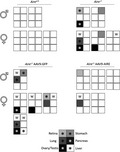

## Introduction

Autoimmune polyglandular syndrome type 1 (APS‐1) also known as autoimmune polyendocrinopathy candidiasis ectodermal dystrophy (APECED) is an incurable monogenic autosomal recessive disorder^1^ classed as a primary immunodeficiency disease (PID). It is characterised by the manifestations of both endocrine and nonendocrine organ destruction.^1^, ^2^ Typically, the initial presentation is with chronic mucocutaneous candidiasis (CMC), followed by hypoparathyroidism and then adrenal insufficiency (Addison’s disease).^1^, ^2^ Serum from APS‐1 patients was found to have high titres of autoantibodies against multiple tissue‐specific antigens (TSAs).^3^ For instance, autoantibodies to the adrenal glands were identified in APS‐1 patients and autoantibodies to NLRP5 were detected in almost 50% of APS‐1 patients with hypoparathyroidism and absent in patients without hypoparathyroidism.^4^ Similarly, autoantibodies to thyroid were detected in APS‐1 patients with hypothyroid who were seropositive to thyroglobulin (TG) and thyroid peroxidase (TPO).^5^ Furthermore, autoantibodies against CYP21A2, CYP17A1 and CYP11A1 were detected in APS‐1 patients with Addison’s disease.^6^, ^7^

Autoimmune polyglandular syndrome type‐1 is caused by loss‐of‐function mutations in the autoimmune regulator (*AIRE*) gene,^8^, ^9^, ^10^ resulting in a dysfunctional or absent AIRE protein. *AIRE* represents the first single‐gene defect resulting in a multisystem autoimmune disease.^11^ To date, more than 100 APS‐1 causing mutations have been identified which vary from substitutions, insertions and deletions to splice‐site mutations.^12^, ^13^, ^14^ Several studies have demonstrated that AIRE/Aire is expressed mainly by the thymus, in a subpopulation of medullary thymic epithelial cells (mTECs), AIRE^+^ mTECs.^9^, ^15^, ^16^, ^17^ AIRE/Aire promotes self‐tolerance in the thymus by regulating the promiscuous expression of a wide array of tissue‐specific antigens (TSAs).^18^ Several studies demonstrated that TSA expression levels reflect Aire expression pattern in a dose‐dependent manner.^19^, ^20^, ^21^ In APS‐1 patients, dominant inheritance of heterozygous missense mutations in *AIRE* produces a reduced level of AIRE and is characterised by a milder phenotype.^22^ Similarly, G228W, the autosomal dominant mutation in APS‐1 patients, presents a mild phenotype compared to patients with an autosomal recessive mutation.^23^, ^24^ The G228W‐knock‐in mouse model partially expressed Aire‐dependent TSAs with a disease spectrum with milder autoimmune phenotype.^25^ These findings suggest that precise level of functional Aire protein is crucial for efficient induction of TSA expression and thus negative selection of T cells to avert autoimmunity.

Studies using *in vivo* APS‐1 mouse models have significantly increased our knowledge of AIRE function and APS‐1‐associated autoimmunity.^26^ The first animal model for APS‐1 was engineered in 2002; these *Aire* ^−/−^ mice exhibited lymphocytic infiltration and circulating serum autoantibodies against specific structures of targeted organs.^18^, ^27^ *Aire* ^−/−^ mice have been used to elucidate the function of Aire, and its primary action is to promote promiscuous gene expression of TSAs within mTECs and the clonal deletion of autoreactive T cells.^18^

Gene‐based delivery of AIRE could be an efficient way of treating APS‐1 patients because the current treatment of APS‐1 is challenging and dependent on multiple therapeutic agents that address individual manifestations, such as hormone replacement therapy, antifungal agents and immunosuppressive therapy for severe features such as autoimmune hepatitis, interstitial lung disease, enteropathy and malabsorption associated with exocrine pancreatic insufficiency.^3^, ^28^ In an attempt to compensate for Aire expression in athymic mice, cotransfer of one thymic lobe from each *Aire* ^+/+^ and *Aire* ^−/−^ into athymic mice did not prevent autoimmunity.^29^ However, when one thymic lobe from *Aire* ^−/−^ and 4 thymic lobes from *Aire* ^+/+^ were transferred into athymic mice, autoimmune infiltrates were reduced, suggesting a gene dose effect.^29^

Gene therapy offers a window of opportunity to tackle monogenic disorders such as APS‐1. Multiple animal studies have been successfully undertaken using viral vectors, providing optimism for the future utility of viral gene delivery as a therapeutic strategy for various conditions.^30^, ^31^, ^32^ Currently, the adeno‐associated (AAV)‐based system is one of the most refined and effective gene delivery systems for multiple cell types. Several reports have shown that AAV serotype 9 (AAV9) mediates efficient and sustained transgene expression following systemic administration in neonatal mice, which was sufficient to extend survival in mouse models of spinal muscular atrophy (SMA).^31^, ^33^ Remarkable safety and efficacy data were reported from 2 separate phase I/II clinical trials in haemophilia B and SMA patients.^34^, ^35^, ^36^

This study elucidates the therapeutic potential of AAV9‐mediated AIRE gene replacement in *Aire* ^−/−^ mice. Because of the monogenic aetiology of APS‐1, we hypothesised that the thymic compartment can be targeted to modulate immune tolerance by using a gene therapy approach to restore a functional copy of the *AIRE* gene. Our study revealed that gene delivery using AAV9 vectors (AAV9‐AIRE) into mouse thymus led to a profound amelioration of the APS‐1 phenotype in *Aire* ^−/−^ mice.

## Results

### Characterisation of the Aire Null phenotype in mice

**Figure 1:**
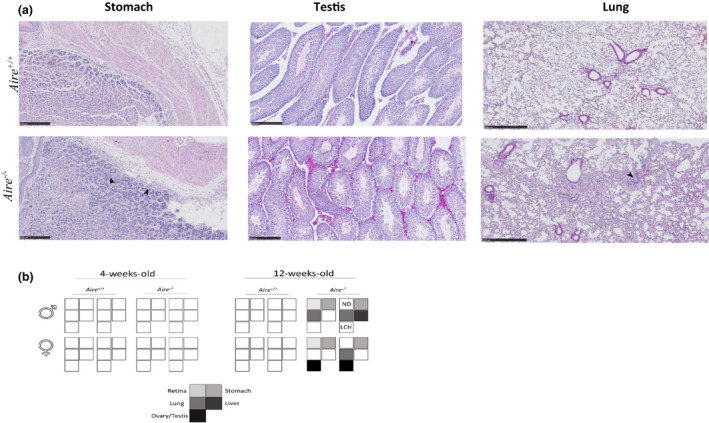
Tissue lymphocytic infiltration of 12‐week‐old Aire^−/−^ mice. **(a)** Tissue section of stomach, testis and lung of 12‐week‐old Aire^+/+^ and Aire^−/−^ mice was stained with haematoxylin and eosin (H&E). Lymphocyte infiltration of stomach, testis and lung of Aire^−/−^ mice is indicated by arrows. Scale bar = stomach, 250 μm; testis, 250 μm; lung, 500 μm. **(b)** Summary of tissue infiltrate in *Aire* ^+/+^ and *Aire^−/−^* mice. In 4‐week‐old mice, no tissue infiltration was observed in *Aire* ^+/+^ or *Aire* ^−/−^ mice. In 12‐week‐old mice, tissues with lymphocytic infiltration are highlighted for each *Aire* ^−/−^ mouse.

As the onset of the disease could be a crucial step in determining the efficiency of the treatment, the histopathological phenotype of Aire^−/−^ mice was performed prior to and early in the disease. The tissues of two groups of 4‐ and 12‐week‐old Aire^−/−^ and Aire^+/+^ littermates were analysed. No observable lymphatic infiltrations were detected in 4‐week‐old Aire^−/−^ mice in eyes, lung, liver, stomach or reproductive organs, consistent with previous studies.^18^, ^37^, ^38^, ^39^, ^40^ However, in 12‐week‐old Aire^−/−^ mice, all mice (*n* = 4) exhibited infiltrates in one or several organs including liver, lung, stomach and testis. The latter showed also a Leydig cell hyperplasia (LCH) morphology leading to autoimmune gonadal atrophy (Figure 1a). No observable infiltrations were detected in any tissues in 12‐week‐old Aire^+/+^ mice (Figure 1b).

### Construction and *in vitro* validation of AAV9‐AIRE

**Figure 2:**
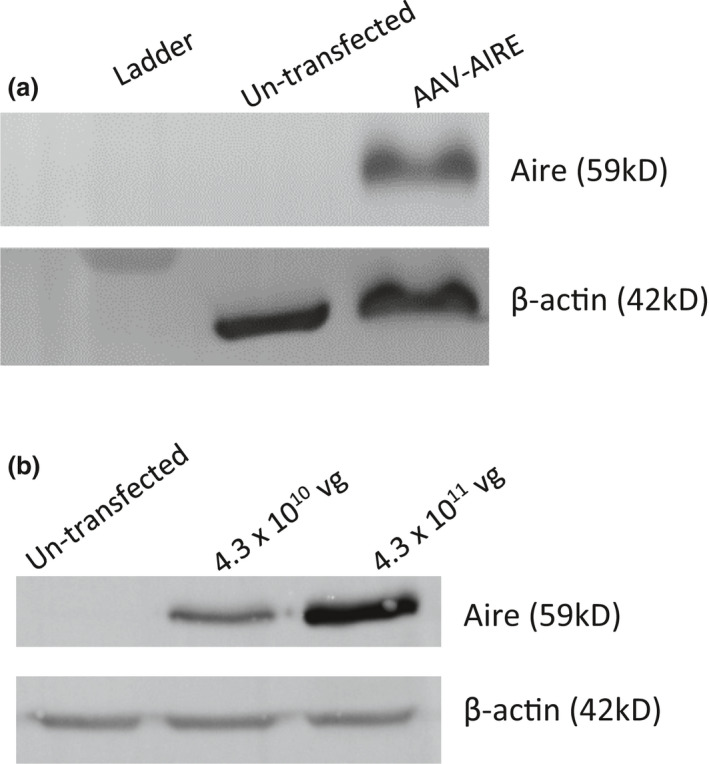
Generation and *in vitro* validation of AAV9‐AIRE. **(a)** Western blot of AIRE expression 48 h after transfection of HEK293 cells with the pAAV‐AIRE plasmid. AIRE levels were normalised to β‐actin levels. **(b)** Western blot of AIRE expression 5 days after transduction of HEK293 cell AAV9‐AIRE viral vector. AIRE levels were normalised to β‐actin levels.

A plasmid harbouring a single‐stranded AAV9 expression cassette carrying the wild‐type human AIRE complementary DNA (cDNA) [National Centre for Biotechnology Information (NCBI) accession number NM_000383.3] coding sequence under the control of the cytomegalovirus (CMV) promoter (AAV‐AIRE) was generated (Supplementary figure 1). We then transfected human embryonic kidney (HEK) 293 cells, which resulted in up‐regulated expression of AIRE protein as assessed by Western blot (Figure 2a). After large‐scale production of the AAV9‐AIRE viral vector, we evaluated its potency to express AIRE by HEK293 cell transduction with 4.3 × 10^10^ and 4.3 × 10^11^ vector genomes (vg) per well followed by Western blotting (Figure 2b). A concentration of 4.38 × 10^10^ vg μL^−1^ of AAV9‐AIRE yielded high AIRE expression level compared to untransduced. In addition, higher expression of AIRE was observed at a concentration of 4.38 × 10^11^ vg μL^−1^. Densitometry analysis confirmed this dose‐dependent pattern of AIRE expression, showing more than twofold increase in AIRE expression with the higher dose at 5 days after transduction as compared to transduced cells with 4.38 × 10^10^ vg μL^−1^ (Figure 2c).

### AAV9‐mediated AIRE expression in the thymi of Aire^−/−^ mice AAV9‐AIRE

To determine the optimal dose of AAV9‐AIRE to be used for our efficacy studies, a dose‐dependent experiment was carried out on Aire mice with the doses of the virus listed in Table 1. Four groups of mice were recruited: *Aire* ^+/+^ mice were injected with PBS (*n* = 5), *Aire^−/−^* were injected with PBS (*n* = 5), *Aire^−/−^* mice were injected with AAV9‐GFP (*n* = 5), and *Aire^−/−^* mice were injected with AAV9‐AIRE (*n* = 5), each mouse with a different dose (Table 1). The total dose of 1.6 × 10^10^ vg was well tolerated by the animal and gave the best response in terms of clearance of circulating autoantibodies and tissue infiltration in these animals.

**Table 1:** Concentrations of AAV9‐AIRE and AAV9‐GFP injected per mouse

Dilution of the viral prep	Amount of virus injected per mouse
1	8 × 10^11^ vg
1:2	4 × 10^11^ vg
1:10	8 × 10^10^ vg
1:50	1.6 × 10^10^ vg
1:100	8 × 10^9^ vg

**Figure 3:**
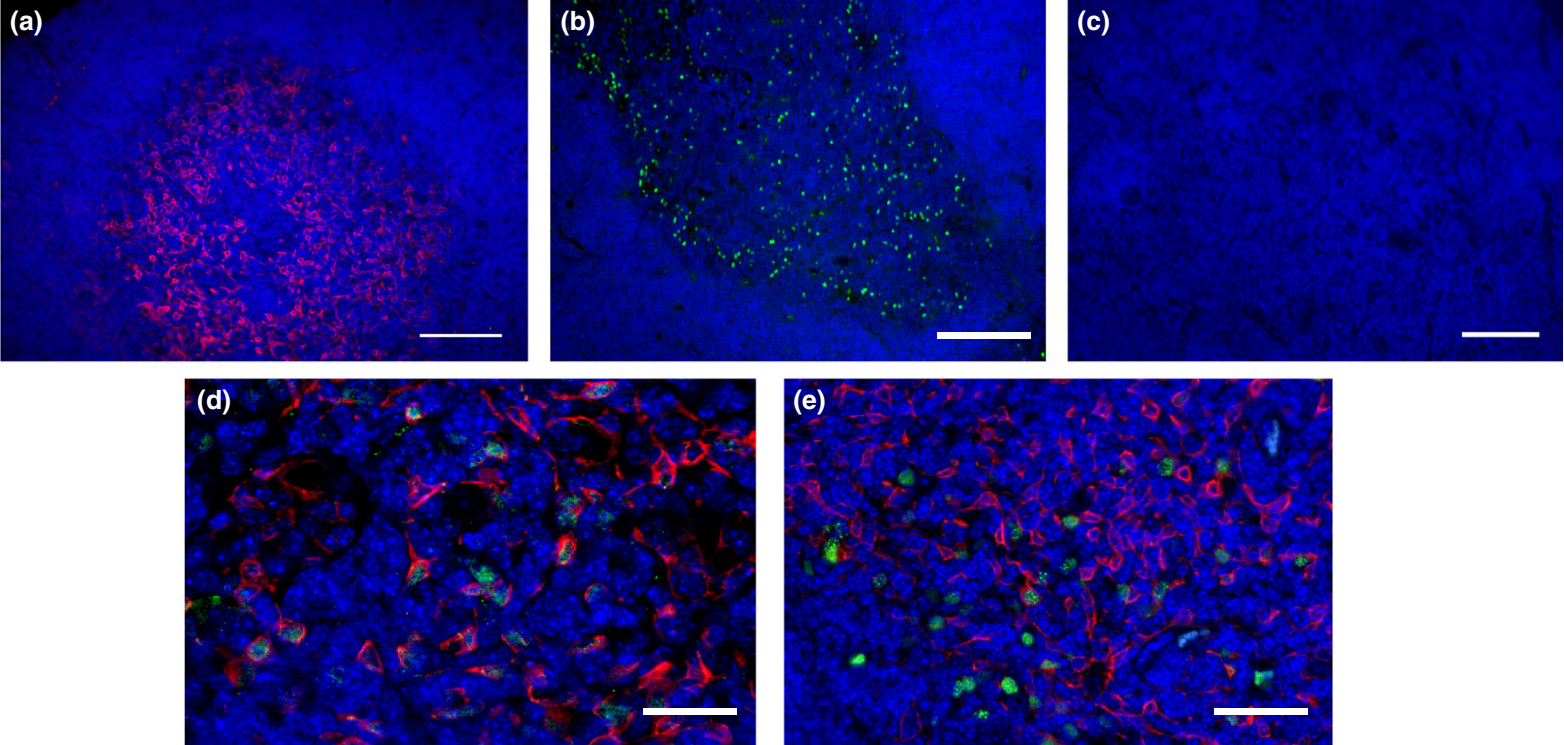
Representative images of transduced thymi of 8‐week‐old APS‐1 mice. Aire^−/−^ mice were injected at 4 weeks old either with PBS, with AAV9‐GFP or with AAV9‐AIRE. Thymi were extracted at 4 weeks postinjection. Thymic sections from mice injected with PBS were labelled with a keratin 5 antibody shown in red colour **(a)**. Thymic sections from mice injected with AAV9‐GFP were stained with a GFP antibody **(b)** or AIRE antibody **(c)**. Thymic sections from mice injected with AAV9‐AIRE were double‐stained with AIRE antibody (green colour) and K5 antibody (red colour) **(d)**. A positive control with staining of thymic sections from wild‐type mice with AIRE antibody (green colour) and K5 antibody (red colour) **(e)**. Primary antibodies coupled to Alexa Fluor 488 secondary antibody and counterstained with DAPI for visualisation of nuclei. AAV9, adeno‐associated virus serotype 9; AIRE, autoimmune regulator; DAPI, diamidino‐2‐phenylindole (blue); GFP, green fluorescent protein; PBS, phosphate‐buffered saline. Scale bar = 100 μm in **a**, 100 μm in **b**, 100 μm in **c**, 50 μm in **d** and 100 μm in **e**.

To examine the transduction efficiency of AAV9 *in vivo*, we injected *Aire* mice at 4 weeks old (before the establishment of the disease) or 8 weeks old (after the establishment of the disease) with AAV9 encoding green fluorescent protein (GFP) (AAV9‐GFP), AAV9‐AIRE or PBS intrathymically at a total dose of 1.6 × 10^10^ vg per mouse. As expected, negative controls represented by mice injected with PBS showed no staining for Aire in the medulla as highlighted by medulla marker K5 staining only (Figure 3a; *n* = 5). Targeting the medulla was a priority for full therapeutic potential; therefore, expression at this site was validated using AAV9‐GFP. Mice injected with AAV9‐GFP led to widespread thymic medulla transduction, as indicated by the fluorescence intensity of GFP in the thymus 4 weeks postinjection (Figure 3b; *n* = 5). As expected, AIRE was not detected in the thymus of mice injected with AAV9‐GFP (Figure 3c; *n* = 5). In contrast, mice injected with AAV9‐AIRE led to AAV9‐AIRE transduced mTECs resulting in AIRE expression within cell nuclei in a punctate pattern (Figure 3d; *n* = 5) similar to endogenous Aire in wild‐type littermates (Figure 3e; *n* = 5). Keratin 5 antibody was used as a surface marker to identify mTECs^41^, ^42^, ^43^ (Figure 3a; *n* = 5).

### AIRE gene replacement restores Aire‐dependent TSAs in the thymus

**Figure 4:**
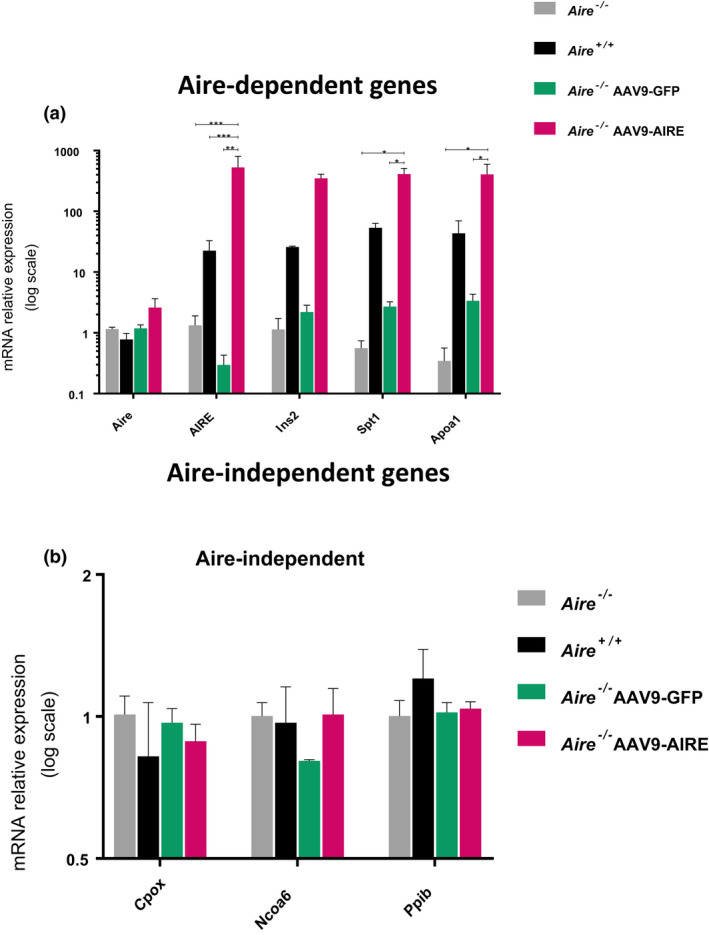
Aire‐dependent and Aire‐independent tissue‐specific antigen (TSA) expression after viral delivery of AIRE *in vivo* in APS‐1 mice. Quantitative PCR analysis of *Aire*/*AIRE* expression and Aire‐dependent including *Ins2*, *Spt1* and *Apoa1* **(a)** and Aire‐independent including *Cpox*, *Ncoa6* and *Ppib* **(b)** TSAs in thymic cells obtained at 4 weeks postinjection from *Aire* ^+/+^ mice (*n* = 5), *Aire* ^−/−^ mice (*n* = 5), *Aire^−/−^* AAV9‐GFP mice (*n* = 5) and *Aire^−/−^* AAV9‐AIRE‐injected mice (*n* = 5). Data are normalised to the expression of *β‐actin* and are presented relative to the expression values in *Aire^−/−^* thymic cells. Data are analysed with one‐way ANOVA, Tukey's multiple comparison test, ****P < 0.0001, **P < 0.005, *P ≤ 0.05. Error bars represent ± SEM. Aire, murine autoimmune regulator; AIRE, human autoimmune regulator; *Apoa1*, apolipoprotein A‐I; *Cpox*, coproporphyrinogen oxidase; *Ins2*, insulin II; *Ncoa6*, nuclear receptor coactivator 6; *Ppib*, peptidylprolyl isomerase B; *Spt1*, salivary protein 1. *Aire* ^−/−^ mice expressed Aire mRNA, but the produced protein is nonfunctional.^18^

To quantify the restored *AIRE* at the mRNA level, qPCR analysis (see Methods) was performed on thymic lysates of all groups. Four weeks postinjection, AAV9‐mediated gene transfer resulted in a significant increase (*P ≤ *0.05) in *AIRE* mRNA levels in thymic cells compared with PBS and AAV9‐GFP injections and a 1000‐fold change compared with endogenous levels in *Aire^−/−^* mice (Aire is expressed but not translated into functional protein in these mice) (Figure 4a; *n* = 3). To determine whether an increase in Aire‐dependent TSAs followed AIRE overexpression, we tested a panel of well‐established Aire‐dependent TSAs including Ccl1, IL‐3, Ins2, Spt1, Apoa1 and Reg1.^29^, ^44^ Indeed, restoration of AIRE levels, mediated by intrathymic AAV injection, resulted in an elevation of expression of all 6 Aire‐dependent TSAs studied (Figure 4a; *n* = 3). A significant increase was observed in both *Ins2* (*P *< 0.0001) and *Spt1* (*P <* 0.005) when compared to PBS‐treated *Aire^−/−^* mice. There was also a significant increase of Ins2 expression in the thymus of AAV9‐AIRE compared to control mice including *Aire^−/−^ mice and* AAV9‐GFP mice (Figure 4a; *n* = 3). In contrast, none of the Aire‐independent genes including Cpox, Ncoa6 and Pplb showed significant changes in the level of their expression between the 4 groups of mice (Aire^−/−^, Aire^+/+^, Aire^−/−^AAV9‐GFP and Aire^−/−^AAV9‐AIRE) (Figure 4b; *n* = 3). These data indicate that modulation of AIRE *in vivo* can directly lead to alterations in specific TSA levels and may have the potential to affect the maintenance of central tolerance.

**Figure 5:**
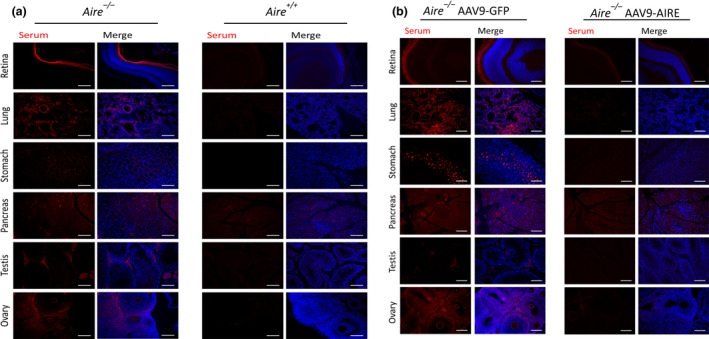
Representative serum autoantibodies in *Aire* ^+/+^, *Aire* ^−/−^, *Aire* ^−/−^ AAV9‐GFP and *Aire* ^−/−^ AAV9‐AIRE mice (8 weeks old). Serum was collected from *Aire* ^+/+^ mice (*n* = 5), *Aire* ^−/−^mice (*n* = 5), *Aire* ^−/−^ AAV9‐GFP mice (*n* = 5) and *Aire* ^−/−^ AAV9‐AIRE mice (*n* = 5) at 4 weeks postinjection. Tissues including retina, lung, stomach, pancreas, testis and ovary were obtained from healthy 8‐week‐old *Aire* ^+/+^ mice. Sections were incubated with individual mouse serum. Nuclei were counterstained with DAPI. AAV9, adeno‐associated virus serotype 9; AIRE, autoimmune regulator; DAPI, diamidino‐2‐phenylindole (blue). Scale bar 100 μm.

To determine whether forced expression of AIRE had maintained the self‐tolerance in *Aire* ^−/−^ mice, we analysed the presence of autoantibodies in *Aire* ^−/−^ mice sera using 4% PFA‐fixed tissue sections from healthy mice by indirect immunofluorescence. In 8‐week‐old mice, circulating autoantibodies against retina, lung, stomach, testis and ovary were similar in *Aire* ^−/−^ mice injected with PBS and *Aire* ^−/−^ mice injected with AAV9‐GFP (Figure 5; *n* = 5). At 8 weeks, serum autoantibodies were found against 1 or 2 tissues (stomach, lung, retina and ovary/testis) per mouse. In *Aire* ^−/−^ mice which were untreated (*n* = 10), 60% exhibited autoimmunity against the retina, ranging from a weak to a strong reaction. In addition, autoantibodies against the reproductive tissues, testis and ovary, were present in 60% of *Aire* ^−/−^ mice. Furthermore, 40% of *Aire* ^−/−^ mice control group displayed autoreactivity against both lung and stomach. In complete contrast, weak or almost undetectable serum reactivity was observed in *Aire* ^+/+^ mice injected with PBS and *Aire* ^−/−^ mice injected with AAV9‐AIRE (Figure 5; *n* = 5). These data suggest that AAV9‐AIRE significantly decreased circulating autoantibodies from the sera of 8‐week‐old *Aire* ^−/−^ AAV9‐AIRE mice.

**Figure 6:**
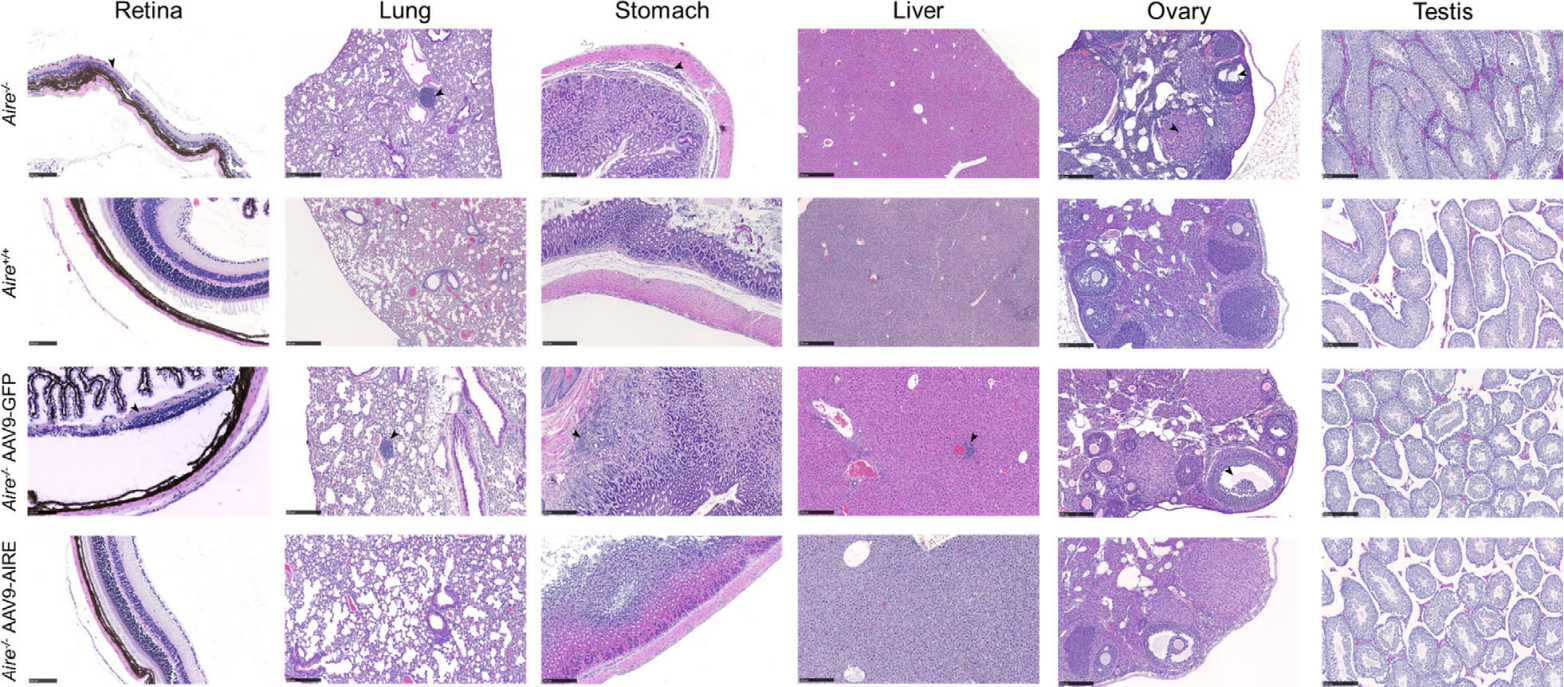
Representative tissue sections from 8‐week‐old APS‐1 mice. Retina, lung, liver and stomach sections from *Aire* ^+/+^, *Aire^−/−^*, *Aire^−/−^* AAV9‐GFP and *Aire^−/−^* AAV9‐AIRE mice, stained with haematoxylin and eosin (H&E). Arrowhead indicates infiltration. Scale bar: retina, 100 μm; liver, 500 μm; lung, stomach, ovary and testis, 250 μm.

**Figure 7:**
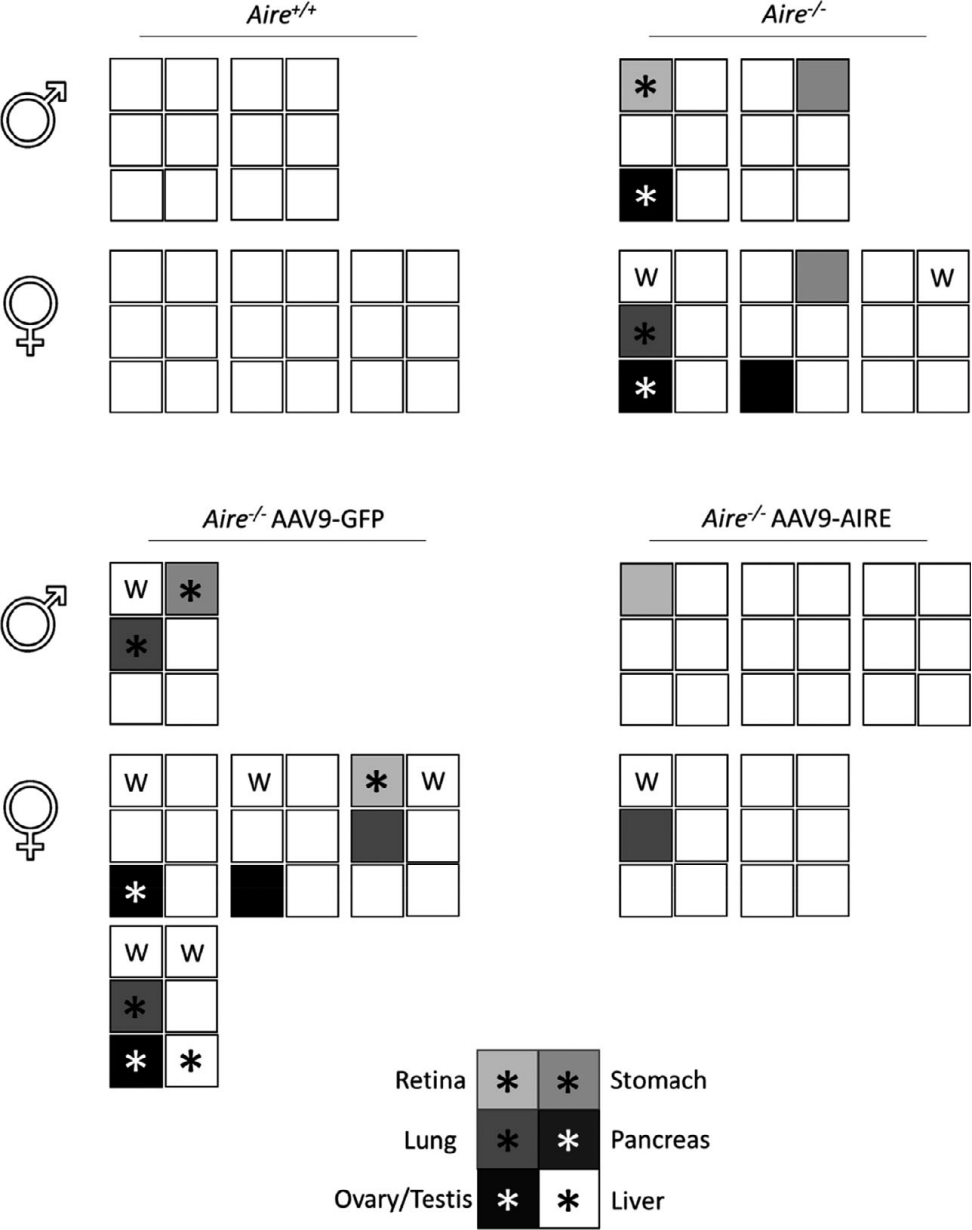
Correlation of serum autoantibodies and tissue infiltrations in 8‐week‐old groups of APS‐1 mouse model (8 weeks old). For each mouse of the indicated group AAV9 virus and gender, the presence (shaded) or absence of autoantibodies against retina, lung, stomach, pancreas and ovary/testis is displayed. An asterisk indicates tissue infiltration.

In the APS‐1 mouse model, infiltrations show an age‐dependent pattern, ranging from none at 4 weeks to at least three or four tissues at 12 weeks.^37^ In keeping with this, at 8 weeks old, retinal degeneration, damaged oocytes and LCH morphology were detected in *Aire^−/−^* mice and AAV9‐GFP‐treated *Aire^−/−^* mice (Figure 6; *n* = 5). Furthermore, mild infiltrations were observed in the lung, stomach and liver of the same mice groups (Figure 6). In agreement with several studies, the majority of *Aire* ^−/−^ mice with tissues targeted by serum autoantibodies are also targeted by lymphocytic infiltrates.^18^, ^37^ All *Aire^−/−^* mice injected with AAV9‐AIRE (*n* = 5) showed no or very minimal tissue damage or infiltration in the investigated tissues (Figure 6; *n* = 5), suggesting that these mice were protected from tissue‐specific autoimmune reactions at this time point. Correlation of serum autoantibodies and infiltrations into retina, lung, stomach, pancreas and ovary/testis tissues is summarised in Figure 7. The latter showed strong correlation between the presence of serum autoantibodies and tissue infiltration. Mice treated with AAV9‐AIRE showed a trace or no circulating autoantibodies and lymphatic tissue infiltration (Figure 7; *n* = 5).

### AAV9‐AIRE gene transfer in Aire‐deficient mice after disease onset

**Figure 8:**
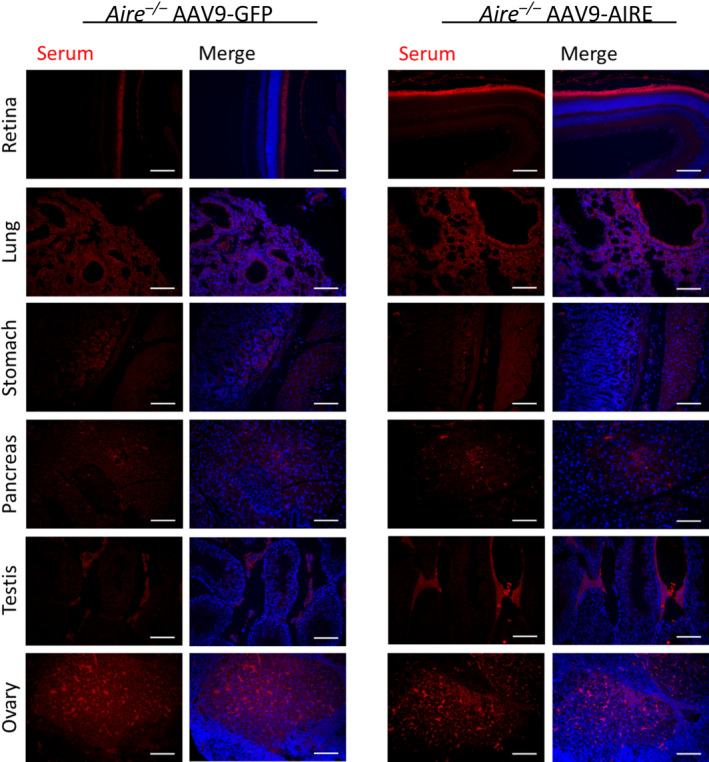
Representative serum autoantibodies in *Aire* ^+/+^, *Aire* ^−/−^, *Aire* ^−/−^ AAV9‐GFP and *Aire* ^−/−^ AAV9‐AIRE mice (16 weeks old). Serum was collected from *Aire* ^+/+^ mice (*n* = 5),* Aire* ^−/−^mice (*n* = 5), *Aire* ^−/−^ AAV9‐GFP mice (*n* = 5) and *Aire* ^−/−^ AAV9‐AIRE mice (*n* = 5) 4 weeks postinjection. Tissues including retina, lung, stomach, pancreas, testis and ovary were obtained from healthy 8‐week‐old *Aire* ^+/+^ mice. Sections were incubated with individual mouse serum. Nuclei were counterstained with DAPI. AAV9, adeno‐associated virus serotype.

**Figure 9:**
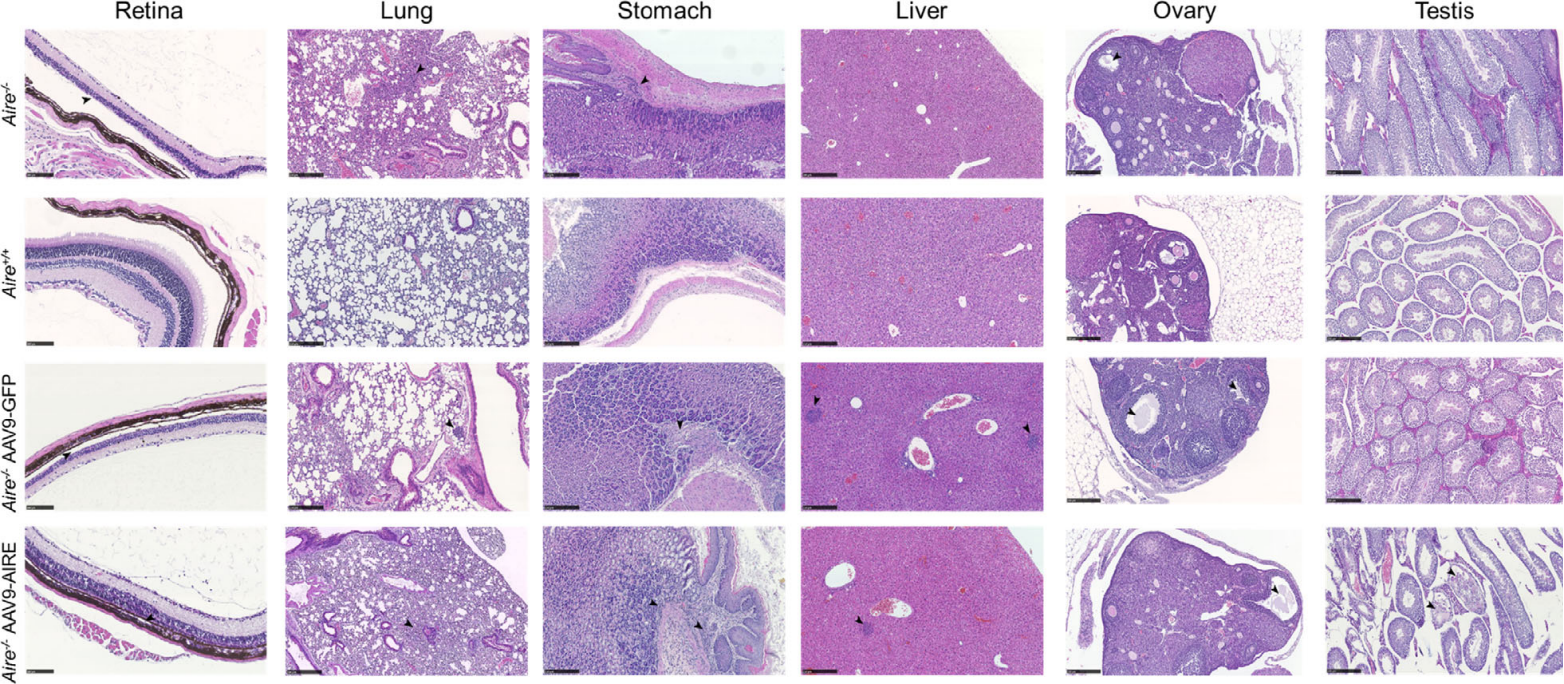
Representative tissue sections from 16‐week‐old APS‐1 mice. Retina, lung, liver and stomach sections from *Aire* ^+/+^, *Aire^−/−^*, *Aire^−/−^* AAV9‐GFP and *Aire^−/−^* AAV9‐AIRE mice, stained with haematoxylin and eosin (H&E). Arrowhead indicates infiltration. Scale bar: retina, 100 μm; liver, 500 μm; lung, stomach, ovary and testis, 250 μm.

**Figure 10:**
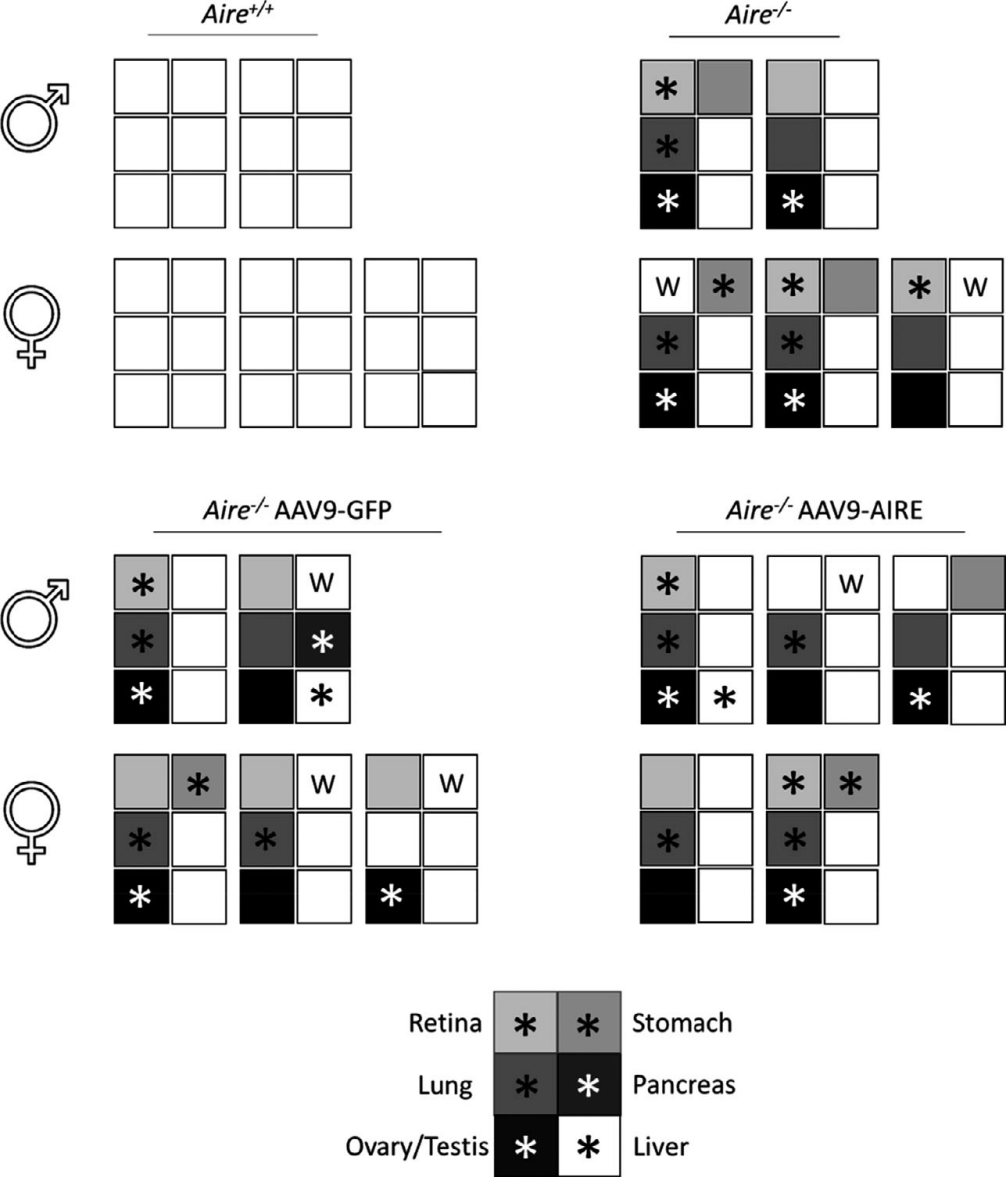
Correlation of serum autoantibodies and tissue infiltrations in 8‐week‐old groups of APS‐1 mouse model (16 weeks old). For each mouse of the indicated group AAV9 virus and gender, the presence (shaded) or absence of autoantibodies against retina, lung, stomach, pancreas and ovary/testis is displayed. An asterisk indicates tissue infiltration.

To determine whether there is rescue of the phenotype in Aire‐deficient mice alongside established autoimmunity after onset of the disease, a group of 8‐week‐old mice (*n* = 5) was intrathymically injected with AAV9‐AIRE 1.6 × 10^10^ vg per mouse. Control groups were injected either with PBS or with AAV9‐GFP. At 4 weeks postinjection, circulating autoantibodies against retina, lung, stomach, testis and ovary were detected in *Aire* ^−/−^ mice injected with PBS and *Aire* ^−/−^ mice injected with AAV9‐GFP (Figure 8; *n* = 5). At 12 weeks of age, serum autoantibodies were found against 1 or 2 tissues per mouse in the investigated tissues. At this later stage of the disease, mice injected with AAV9‐AIRE or AAV9‐GFP showed a similar pattern of reactive autoantibodies. Serum autoantibodies from these mice showed reactivity to several tissues including retina, lung, stomach, testis and ovary of control mice (Figure 9; *n* = 5), suggesting the disease needs to be targeted early with this approach. Correlation of serum autoantibodies and infiltrations into retina, lung, stomach, pancreas and ovary/testis tissues is summarised in Figure 10. The latter showed strong correlation between the presence of serum autoantibodies and tissue infiltration. There was no difference between mice treated with AAV9‐AIRE or AAV9‐GFP in terms of circulating autoantibodies and lymphatic tissue infiltration, suggesting that AAV9‐AIRE treatment has no significant effect on *Aire* ^−/−^ 12‐week‐old mice (Figure 10).

## Discussion

Direct injection of AAV9‐AIRE into 4‐week‐old AIRE KO mice leads to the clearance of circulating autoantibodies and reduced T‐cell infiltration into tissues of these mice. The main rationale behind the choice of AAV9 rather than other serotype is the fact AAV9 has an established clinical and safety profile. Indeed, AAV9 has been used for other clinical trials showing remarkable safety and efficacy outcomes (e.g. spinal muscular atrophy).^31^, ^33^

This proof‐of‐concept efficacy study has demonstrated that a noninvasive procedure of gene delivery with an ultrasound imaging technique is efficient in targeting AIRE to the medulla of the thymus. This is important because it avoids expressing AIRE in thymic epithelium cells which lead to autoimmunity^45^. This study also showed that 4 weeks post‐intrathymic injections of AAV9‐AIRE in *Aire* ^−/−^ mice, mRNA encoding AIRE and Aire‐dependent TSAs were significantly elevated. Correspondingly, following AIRE expression, serum autoantibodies levels were reduced 4 weeks postinjection. The novel molecular therapy approach employed led to an ameliorated autoimmunity phenotype in APS‐1 mouse model.

Modulation of AIRE *in vivo* can directly lead to alterations in TSA levels and may affect the maintenance of central tolerance. To confirm that thymic expression of TSAs does affect self‐tolerance, serum autoantibodies from AAV9‐AIRE‐injected mice were screened for autoreactivity against several tissues including retina, lung, pancreas, stomach, ovary and testis.^18^, ^37^ The investigated sera harboured no autoantibodies against the key tissues. In addition, histological analysis of *Aire* ^−/−^ treated with AAV9‐AIRE revealed no tissue infiltrations at all. Thus, the expected disease phenotype was ameliorated when compared to the autoimmunity profile of *Aire* ^−/−^ mice and AAV9‐GFP‐injected *Aire* ^−/−^ mice.

Several studies have reported that Aire promotes the generation of a distinct repertoire of T_reg_ during the perinatal period that stably persists in adult mice.^46^, ^47^, ^48^ When *Aire* was turned on by doxycycline during the neonatal period up to 3 weeks after birth and then turned off, these mice were protected from autoimmunity.^46^ Following this, several investigations led to the discovery of a distinct subset of T_reg_ produced during the perinatal window and this population has a role in maintaining self‐tolerance unique from those of T_reg_ repertoire produced during adulthood.^47^, ^48^ This highlights the importance of Aire expression early in life in order to maintain long‐term self‐tolerance. However, the overexpression of AAV9 Aire after the perinatal window and during adulthood seems to be essential to produce a T_reg_ repertoire that could ameliorate autoimmunity.

Our molecular therapy study shows that AIRE was expressed in the medulla of thymus. The question that arises as a result is what cells within the thymus are the ultimate targets of AAV9 and which of these drive long‐term expression of the transgene? We suggest that mTEC cells are targeted by AAV9 and that the use of AIRE promoter would restrict AIRE expression to these cells. This approach would be challenging because tissue‐specific expression of AIRE is exclusively controlled by an upstream enhancer region.^49^, ^50^ A potential limitation in translating our findings is that mTEC cells are short‐lived with estimated lifespan of 2–3 days, and since AAV is a nonintegrating vector, the expression and negative T‐cell selection occurring after transduction would be transient. However, our data suggest that transient expression of AIRE in mTEC would be sufficient to confer benefit to patients even if AAV9‐encoded AIRE was expressed transiently and if the disease is targeted early. We propose therefore from our data that there would be specific benefit for paediatric patients since we were able to rescue the AIRE‐deficient phenotype in the early phase of the disease versus later time points. This could be because the subset of T_reg_ cells that protect the organism against potential immune attack is produced early in life, during the perinatal window. Thus, a molecular therapy approach could offer a potential treatment for APS‐1 patients at early age of onset of the disease.

Based on the promising data obtained in our proof‐of‐concept preclinical study, we plan to take our gene therapy approach towards translational path by completing the following tasks: (1) generation of the final configuration of the therapeutic vector; (2) preparation of preclinical safety package and clinical design for discussion with regulators; (3) complete GMP manufacturing of clinical‐grade vector for use in clinical trials; and (4) prepare clinical trial application (CTA).

## Methods

### Mice

B6.129S2‐*Aire^tm1.1Doi^/J* were purchased from the Jackson Laboratory (004743) (The Jackson Laboratory, Bar Harbor, ME, USA). Aire^−/−^mice carry a deletion of exon 2 which causes frame shift and gives an early truncated Aire protein at amino acid 48 out of 545.^18^ All mice were maintained in a controlled facility in a 12‐h dark–light photocycle with free access to food and water and maintained in a specific pathogen‐free animal facility.

### AAV9‐AIRE viral vector production

Single‐stranded AAV (ssAAV) expressing human AIRE cDNA was constructed by ligation of the human AIRE cDNA polymerase chain reaction (PCR) product (forward primer: 5′‐GATGCCGCTAGCGCCGCCACCATGGCGA‐3′; reverse primer: 5′‐CCGGCCAAGCTTGGGCCCTCAATGATG‐3′). NOTE: this was amplified from a previously cloned plasmid in our laboratory. The human AIRE shares 89% amino acid identity with murine Aire. The full CMV promoter was derived from pAAV‐MCS (Stratagene, Stockport, UK). The GFP cDNA of the AAV9‐GFP plasmid was replaced with the AIRE cDNA PCR product. Standard cloning procedures for DNA extraction and production of the large‐scale plasmids were followed using QIAGEN kits following the manufacturer's instructions. High‐titre scAAV9 vectors were then prepared using a three‐plasmid transient cotransfection system. HEK293T cells in 15‐cm dishes were transfected with packaging plasmids pHelper (Stratagene), pAAV2/9 and the transgene vector ssAAV‐AIRE at 2:1:1 ratio, respectively, using polyethylenimine (1 mg mL^−1^) in serum‐free Dulbecco's modified Eagle's medium (DMEM). At 5 days post‐transfection, supernatant containing cell‐released virus was harvested, treated with benzonase (10 unit mL^−1^; Sigma, Poole, UK) for 2 h at 37°C and concentrated to equal approximately 24 mL using Amicon Ultra‐15 Centrifugal 100K Filters (Millipore, Watford, UK). Iodixanol gradient containing 15%, 25%, 40% and 54% iodixanol solution in phosphate‐buffered saline (PBS)/1 mmol L^−1^ MgCl_2_/2.5 mmol L^−1^ KCl and virus solution was loaded and centrifuged at 69 000 revolutions per minute for 90 min at 18°C. After ultracentrifugation, the virus fractions were visualised on a 10% polyacrylamide gel, stained using SYPRO Ruby (Life Technologies, Paisley, UK) according to the manufacturer's guidelines. The highest purity fractions (identified by the presence of the three bands corresponding to VP1, VP2 and VP3) were pooled and concentrated further in the final formulation buffer consisting of PBS supplemented with an additional 35 mmol L^−1^ NaCl using Amicon Ultra‐15 Centrifugal 100K Filters. Viral titres were determined by quantitative PCR assays using primers directed against AIRE and a linearised ssAAV‐CMV‐AIRE vector as a standard curve.

### Intrathymic injections

#### Ultrasound imaging of the thymus

To avoid using procedures that require opening the thorax and to improve success rates with noninvasive procedures, ultrasound imaging technique^51^, ^52^ was adopted for accurately locating the thymus in anaesthetised 4‐week‐old mice. To validate this technique, ten 4‐week‐old Aire mice were used. Using the Vevo 770 (VisualSonics, Toronto, Canada) preclinical scanner, the thymus of 4‐week Aire mice could easily be visualised. Syringe needle within the thymus was detected by the ultrasound imaging system. Four groups of mice were recruited: *Aire* ^+/+^ mice were injected with PBS (*n* = 5), *Aire^−/−^* were injected with PBS (*n* = 5), *Aire^−/−^* mice were injected with AAV9‐GFP (*n* = 5), and *Aire^−/−^* mice were injected with AAV9‐AIRE (*n* = 5).

#### Serum autoantibody screening

Mice were killed at 4 weeks post‐vector delivery. Designated tissues were removed, fixed in 4% PFA and embedded in paraffin. Tissue sections were stained with H + E, and infiltration of various organs was scored. The detection of autoAbs was monitored. Briefly, serum was collected from *Aire* ^+/+^ mice injected with PBS, *Aire^−/−^* injected with PBS, *Aire^−/−^* mice injected with AAV9‐GFP and *Aire^−/−^* mice injected with AAV9‐AIRE. Tissues including retina, lung, stomach, pancreas, testis and ovary were obtained from healthy 8‐week‐old *Aire* ^+/+^ mice. Sections were incubated with individual mouse serum. Serums and tissue sections from each animal were anonymously coded to allow blinded accession of each animal.

### Histological analysis

#### Tissue collection

Mice were euthanised by intraperitoneal injection of 500 mg kg^−1^ of sodium pentobarbital (sodium pentobarbital, 20% w/v solution for injections, JML). Tissues were collected and immediately fixed by 4% paraformaldehyde (4% PFA) overnight at 4°C. The tissues were then transferred to PBS, paraffin‐embedded and sectioned at 5 µm. To analyse AAV9‐GFP transduction efficiency, tissues were embedded in OCT and frozen at −80°C. After sectioning, 1 in 10 slides were taken throughout each tissue for analysis. Sections were incubated in xylene twice for 10 min before being hydrated through 100%, 95% and 70% ethanol. After blocking endogenous peroxidase activity and antigen retrieval, sections were incubated with protein block serum‐free (DAKO) for 10 min to block any nonspecific binding. After tissue permeabilisation with 0.3% Triton X‐100, sections were incubated with a polyclonal goat antibody against Aire D‐17 (sc‐17986; Santa Cruz Biotechnology, Heidelberg, Germany) at dilution of 1:50 in 0.15% Triton X‐100 in PBS for 1 hr at room temperature. For serum autoantibody analysis in mouse injected with AAV9‐AIRE, mouse serum was tested using 4% fixed tissue sections from healthy mice by indirect immunofluorescence. We used 15 tissue sections analysed per tissue per animal. For GFP visualisation, AAV9‐GFP thymic sections were fixed with acetone for 20 min and mounted using VECTASHIELD Antifade Mounting Medium with DAPI (Vector Lab, Burlingame, CA, USA). Images were taken with fluorescence microscope (Leica AF6000).

### Quantitative PCR

Total RNA was extracted using the TRI Reagent^®^ (Sigma‐Aldrich, Dorset, UK). Tissues were homogenised using mortar and pestle with liquid nitrogen. TRI Reagent was added on the powdered tissues and transferred into 1.5‐mL Eppendorf tubes for subsequent phase separation. The aqueous phase containing the RNA was separated and transferred to a fresh tube, and then, the RNA was precipitated by adding 0.5 mL of isopropanol per 1 mL of TRI Reagent. Pelleted RNA was then washed with 75% ethanol and resuspended in 30 μL of RNase‐free water. RNA concentration was measured by NanoDrop. RNA was reverse‐transcribed, and cDNA was synthesised using SuperScript^®^ IV First‐Strand Synthesis System Kit by random hexamer (18091050; Invitrogen, Paisley; UK) according to the manufacturer’s protocol. Samples were used for quantitative RT‐PCR (qPCR). qPCR, consisted of 10 μL *power* SYBR™ Green PCR Master Mix (4367659; Applied Biosystems, Paisley, UK), cDNA and 600 nm forward and reverse primers (Supplementary table 1) to a total volume of 20 μL, was run in triplicate. The PCR was carried out in 384‐well plate using 7900HT Real‐Time PCR System (Applied Biosystems).

### Western blots

Cells were washed with cold PBS and pelleted in 1.5‐mL Eppendorf tubes before adding the radioimmunoprecipitation assay (RIPA) buffer which is composed of 50 mm Tris‐HCl pH 7.4 (Sigma‐Aldrich), 150 mm NaCl (Sigma‐Aldrich), 2 mm (ethylenediaminetetraacetic acid) EDTA, 1% Triton X‐100 (Sigma‐Aldrich), 0.5% sodium deoxycholate and 0.1% sodium dodecyl sulphate (SDS) (Sigma‐Aldrich), supplemented with 1% protease inhibitor cocktail (Sigma‐Aldrich). Briefly, the cell pellet was suspended in 200–500 μL of RIPA and incubated for 20 min on ice to lyse the cells. The lysates were centrifuged at 13 000 *g* and 4°C for 10 min, and then, the supernatant containing the protein was separated from cell debris and collected into a labelled 1.5‐mL Eppendorf tube. Before loading the protein, samples were denatured by heating at 95°C for 5 min with Laemmli buffer (Sigma‐Aldrich) and then loaded onto a 10% SDS‐PAGE gel. The gels were electrophoresed in Tris/glycine/SDS running buffer at 60 V for 20 min and then 60–90 min at 120 V. For immunodetection, proteins were transferred onto a PVDF membrane (Millipore). The PVDF membrane was soaked in 100% methanol before being immersed in cold transfer buffer. The proteins were transferred onto the PVDF membrane in transfer buffer at 250 mA for 1 h. Membranes were then blocked in 5% milk TBS‐T blocking buffer for 60 min at room temperature in a roller (Milton). Primary and secondary antibody solutions were prepared in 5% milk TBS‐T blocking buffer. Goat primary antibody against AIRE (sc‐17986; Santa Cruz Biotechnology) was diluted at 1:2500, and rabbit primary antibodies against β‐actin (ab49900, Abcam; Cambridge, UK) and GAPDH (ab181602; Abcam) were diluted at 1:3000 and 1:10 000, respectively. All primary antibodies were incubated at 4°C overnight with agitation. HRP (horseradish peroxidase) secondary antibodies, anti‐goat HRP antibody (P0160; DAKO, Ely, UK) and anti‐rabbit HRP antibody (7074S; Cell Signalling), were used at a dilution of 1:5000 and 1:10 000, respectively, incubated for 1 h at room temperature in a roller (Milton). Between the primary and secondary antibody incubations, the membranes were washed three times for 10 min in TBS‐T at room temperature in the roller (Milton) to remove any unbound antibodies. The proteins were visualised using the ECL Plus Chemiluminescence Detection Kit (GE Healthcare, Little Chalfont, UK) for HRP. The chemiluminescence was visualised using the G:BOX Image Capture System (G:BOX; Syngene).

### Statistical analysis

All the data shown in the present study are reported as means ± SEM. The number of sampled units, *n*, upon which we reported statistic, is the single mouse for the *in vivo* experiments (one mouse is *n* = 1). GraphPad Prism 6 software (GraphPad Software) was used for statistical analyses. *P* < 0.05 was considered significant. For all the data sets, data were analysed by parametric tests, *a* = 0.05 (one‐way ANOVA with Tukey’s post hoc test). The statistical analysis performed for each data set as compared to untreated controls is indicated in figure captions. For all figures, *P < 0.05, **P < 0.01, ***P < 0.001, ****P < 0.0001.

## Conflict of interest

Authors have no conflict of interest to declare.

## Author contributions

**Sarah Almaghrabi:** Data curation; Formal analysis; Funding acquisition; Methodology; Project administration; Writing‐original draft. **Mimoun Azzouz:** Data curation; Formal analysis; Methodology; Supervision; Writing‐review & editing. **Rachid Tazi Ahnini:** Conceptualization; Data curation; Funding acquisition; Investigation; Methodology; Project administration; Resources; Supervision; Validation; Visualization; Writing‐original draft; Writing‐review & editing.

## Ethical approval

All *in vivo* experimental work was approved by local ethic committee and performed in accordance with the UK Home Office Animals (Scientific Procedures) Act 1986 under Animal licence (P815DIFB3).
