# Supplementary material for: AAV9‐mediated AIRE gene delivery clears circulating antibodies and tissue T‐cell infiltration in a mouse model of autoimmune polyglandular syndrome type‐1
Source: Clin Transl Immunology. 2020 Sep 3;9(9):e1166. doi: 10.1002/cti2.1166 (PMC7507015; doi:10.1002/cti2.1166)
Supplement: Supplementary file 1 [file CTI2-9-e1166-s001.docx]

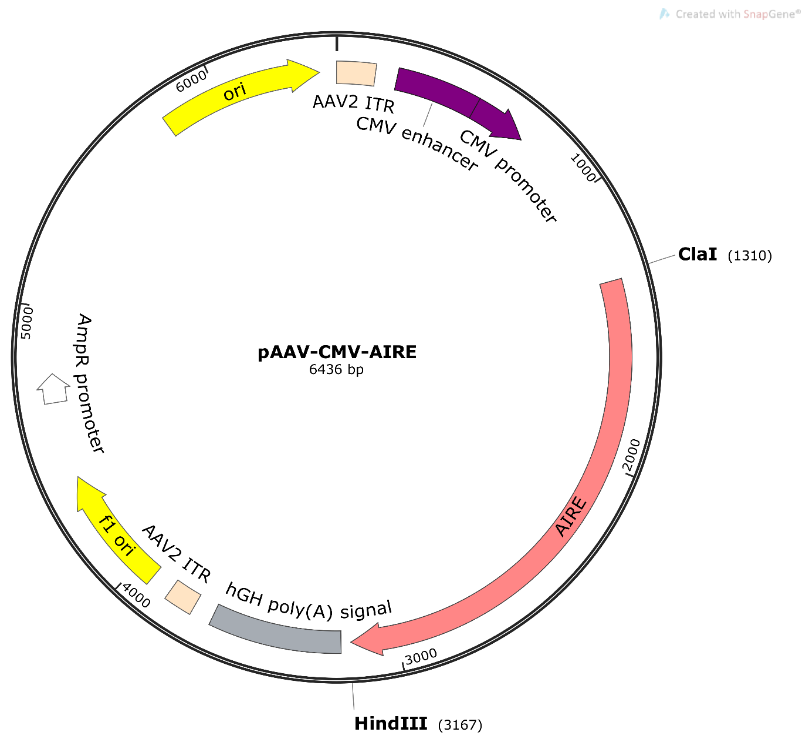


**Supplementary figure 1.** Schematic of pAAV-AIRE vector map. Plasmid carrying AIRE and the CMV promoter. AAV2 inverted terminal repeats appear in flanking positions.
