# Supplementary material for: AAV9‐mediated AIRE gene delivery clears circulating antibodies and tissue T‐cell infiltration in a mouse model of autoimmune polyglandular syndrome type‐1
Source: Clin Transl Immunology. 2020 Sep 3;9(9):e1166. doi: 10.1002/cti2.1166 (PMC7507015; doi:10.1002/cti2.1166)
Supplement: Supplementary file 2 [file CTI2-9-e1166-s002.docx]

| Supplementary table 1. Q-PCR primer sequences | | | |  |
| --- | --- | --- | --- | --- |
| cDNA | **UCSC code** | **Primer sequence** | **Location within cDNA** | **Size (bp)** |
| Aire | uc007fww.2 | For 5’CAGCAACTCTGGCCTCAAAG 3’  Rev 5’CTTCGAACTTGTTGGGTGTATAA 3’ | 518-802 | 285 |
| AIRE | uc062arj.1 | For 5’AGGCAACAGTCCAGGAGGTG 3’  Rev 5’TAGGGGTTCCCCAGGTGGAC 3’ | 1755-1885 | 131 |
| Ccl1 | uc007kmu.1 | For 5’GGCTGCCGTGTGGATACAG 3’  Rev 5’AGGTGATTTTGAACCCACGTTT 3’ | 107-325 | 219 |
| Fabp2 | uc008rex.2 | For 5’GTGGAAAGTAGACCGGAACGA 3’  Rev 5’CCATCCTGTGTGATTGTCAGTT 3’ | 359-475 | 117 |
| IL-3 | uc007ixn.1 | For 5’GGGATACCCACCGTTTAACCA 3’  Rev 5’AGGTTTACTCTCCGAAAGCTCTT 3’ | 124-262 | 139 |
| Ins2 | uc009kog.3 | For 5’GCTTCTTCTACACACCCATGTC 3’  Rev 5’AGCACTGATCTACAATGCCAC 3’ | 231-377 | 147 |
| Spt1 | uc029svn.1 | For 5’CTGGTGAAAATACTGGCTCTGAA 3’  Rev 5’AGCAGTGTTGGTATCATCAGTG 3’ | 178-293 | 116 |
| Csnα | uc029viy.2 | For 5’ACCTTACTCCCAAAGCTGTCCTTA 3’  Rev 5’GAGGGTCCAGTCACATCAAATGT 3’ | 870-1005 | 136 |
| Apoa1 | uc009phb.3 | For 5’ GGCACGTATGGCAGCAAGAT 3’  Rev 5’ CCAAGGAGGAGGATTCAAACTG 3’ | 182-310 | 129 |
| Fam25c | uc007tat.1 | For 5’GAGCAGTTCACGCAGTGGAA 3’  Rev 5’GCATGGGTAACAGCATCAGTG 3’ | 103-274 | 172 |
| Ctrb1 | uc009nms.2 | For 5’ATGGCATTCCTTTGGCTTGTG 3’  Rev 5’GGATAGCATCCTCTCCGTTGAC 3’ | 19-142 | 124 |
| Maoa | uc009ssa.2 | For 5’GCCCAGTATCACAGGCCAC 3’  Rev 5’CGGGCTTCCAGAACCAAGA 3’ | 161-277 | 117 |
| Pitpnc1 | uc011ygq.1 | For 5’CAACCCATCATGTGCTCCTAC 3’  Rev 5’CCCGAACATCATCCATTGTCAT 3’ | 1310-1484 | 175 |
| Riok2 | uc008ape.2 | For 5’TAAGCTGTTCAACAATCCCTCC 3’  Rev 5’GCTGCTTGGTAAACACATTGG 3’ | 1730-1855 | 126 |
| Tmem241 | uc008ebx.2 | For 5’TCTGCACCTGTTACCTGGCT 3’  Rev 5’AATGTCTGCCACCCTTGGAAT 3’ | 120-216 | 97 |
| Cnnm2 | uc008hue.1 | For 5’AAGTGGCCCACCGTGAAAG 3’  Rev 5’CGCTTCTACTTCTGTTGCTAGG 3’ | 1951-2078 | 128 |
| Cpox | uc007zoa.2 | For 5’ACGGGCGTGTGTTTGAAAAG 3’  Rev 5’CACAGAACTTACACCCATAGCAG 3’ | 774-925 | 152 |
| MIIt11 | uc033hxa.1 | For 5’TAAGTAGCCAGTACAGCTCCTT 3’  Rev 5’CGTAGGTAGGTGTATCTGACAGG 3’ | 14-115 | 102 |
| Ncoa6 | uc008nkm.2 | For 5’GAATGTGCCCAACTTGTTACAC 3’  Rev 5’CCCTTCAATCTGAACGGAGAGAA 3’ | 353-536 | 184 |
| Ppib | uc009qei.1 | For 5’GGCTCCGTCGTCTTCCTTTT 3’  Rev 5’ACTCGTCCTACAGATTCATCTCC 3’ | 155-276 | 122 |
| Fezf2 | uc007sfs.2 | For 5’ACTCGGCCTTGACAGCTGAACG 3’  Rev 5’TGAGCATTGAACACCTTGCCGCAC 3’ | 1063-1183 | 121 |
| Foxn1 | uc007kjc.2 | For 5’TTCCATCAGTACTCCCCGGGTGG 3’  Rev 5’GCGTTGGCCTGGGGTGCAAT 3’ | 831-925 | 95 |
| β-actin | uc009ajk.2 | For 5’GGCTGTATTCCCCTCCATCG 3’  Rev 5’CCAGTTGGTAACAATGCCATGT 3’ | 193-346 | 154 |
